# Modeling and NMR Data Elucidate the Structure of a G-Quadruplex–Ligand Interaction for a Pu22T-Cyclometalated Iridium(III) System

**DOI:** 10.1021/acs.jpcb.4c06262

**Published:** 2024-11-19

**Authors:** Carly R. Reed, Scott D. Kennedy, Rachel H. Horowitz, Anees Mohammed Keedakkatt Puthenpeedikakkal, Harry A. Stern, David H. Mathews

**Affiliations:** † Department of Chemistry and Biochemistry, SUNY Brockport, Brockport, New York 14420, United States; ‡ Department of Biochemistry & Biophysics and Center for RNA Biology, University of Rochester Medical Center, Rochester, New York 14642, United States; § Orogen Therapeutics, 12 Gill Street Suite 4200, Woburn, Massachusetts 01801, United States

## Abstract

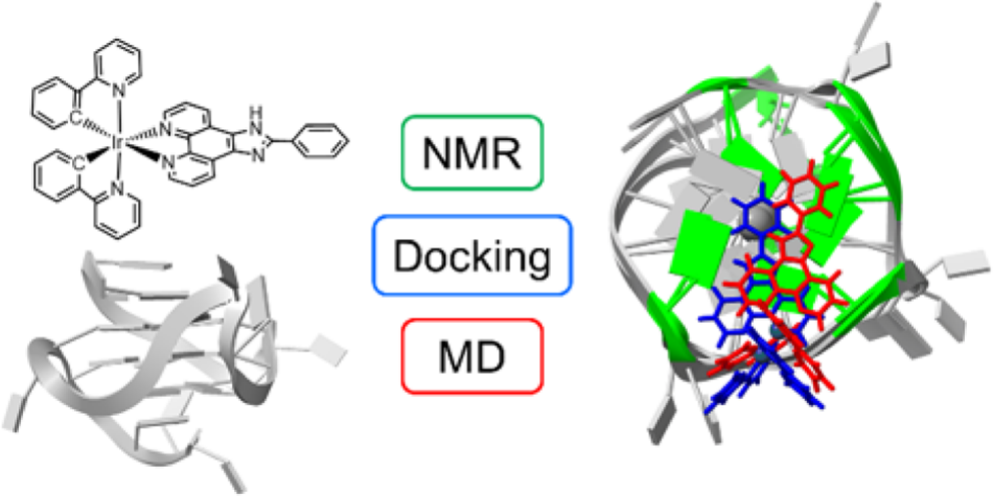

Cyclometalated iridium(III) complexes are increasingly being developed for application in G-quadruplex (GQ) nucleic acid biosensors. We monitored the interactions of a GQ structure with an iridium(III) complex by nuclear magnetic resonance (NMR) titrations and subsequently compared the binding site inferred from NMR with binding positions modeled by molecular docking and molecular dynamics simulations. When titrated into a solution of G-quadruplex **Pu22T**, compound **1(PF**_**6**_), [Ir(ppy)_2_(pizp)](PF_6_), where ppy is 2-phenylpyridine and pizp is 2-phenylimidazole[4,5f][1,10]phenanthroline, had the greatest impact on the hydrogen chemical shifts of G5, G8, G9, G13, and G17 residues of **Pu22T**, indicating end-stacking at the 5′ tetrad. In blind cross-docking studies with Autodock 4, end-stacking at the 5′ tetrad was found as the lowest energy binding position. AMBER molecular dynamics simulations resulted in a refined binding position at the 5′ tetrad with improved pi stacking. For this model system, **Pu22T-1**, molecular docking and molecular dynamics simulations are tools that are able to predict the experimentally determined binding position.

## 1 Introduction

G-quadruplex nucleic acid structures have garnered recognition in the field of biosensing in recent years. This attention is attributed to their cost-effectiveness, easy modification, stability, and ability to act as either the target recognition component or the signal transducer within a biosensor.^1−5^ G-quadruplexes are secondary structures that form in guanine-rich strands of DNA or RNA where tetrads of guanines are held together by Hoogsteen hydrogen bonding and stabilized by monovalent cations.^6^

In the context of GQ biosensors, cyclometalated iridium(III) complexes have emerged as luminescent signaling agents.^7−9^ Like ruthenium(II) complexes, the luminescence of iridium(III) complexes, which is partially or fully quenched in aqueous solution, is turned-on or enhanced when protected from water through nucleic acid binding.^9−12^ The present advantages over organic and other transition metal luminophores include strong, long-lived phosphorescence; high photostability; and selectivity over other forms of nucleic acids.^7,8,13,14^ Structural changes to the iridium complex and GQ sequence and topology impact the binding affinity and luminescence enhancement of signaling agents.^15−18^ Hence, exploring the binding position and structural interactions between iridium signaling agents and GQs is crucial for understanding ligand specificity and influencing future ligand design.

For purely organic ligands, fused polycyclic ligands with planar structures target the 3′ and 5′ terminal G-quartets of unimolecular GQs via tetrad stacking, while flexible ligands and flexible side-chains prefer groove-binding.^19,20^ In cases where metal-containing ligands bind to GQs, 3′ and 5′ terminal tetrad stacking predominately occurs, with a few examples of intercalation.^20,21^ However, the binding of cyclometalated iridium (III) complexes with GQs remains less well understood, with no solution or solid-state structures confirming the binding position of complexes of the general structure, [Ir(N^C)_2_(N^N)](PF_6_), with any GQs. Luminescence studies have indicated a dependence on G-quadruplex loop size, irrespective of loop position.^22^ Docking and molecular dynamic simulations of similar ruthenium complexes predict both tetrad stacking and loop binding, leading to hypotheses that suggest binding interactions may occur in regions where the G-quadruplex loops can protect the iridium(III) complex from the surrounding buffer solution.^16,23^

**Figure 1:**
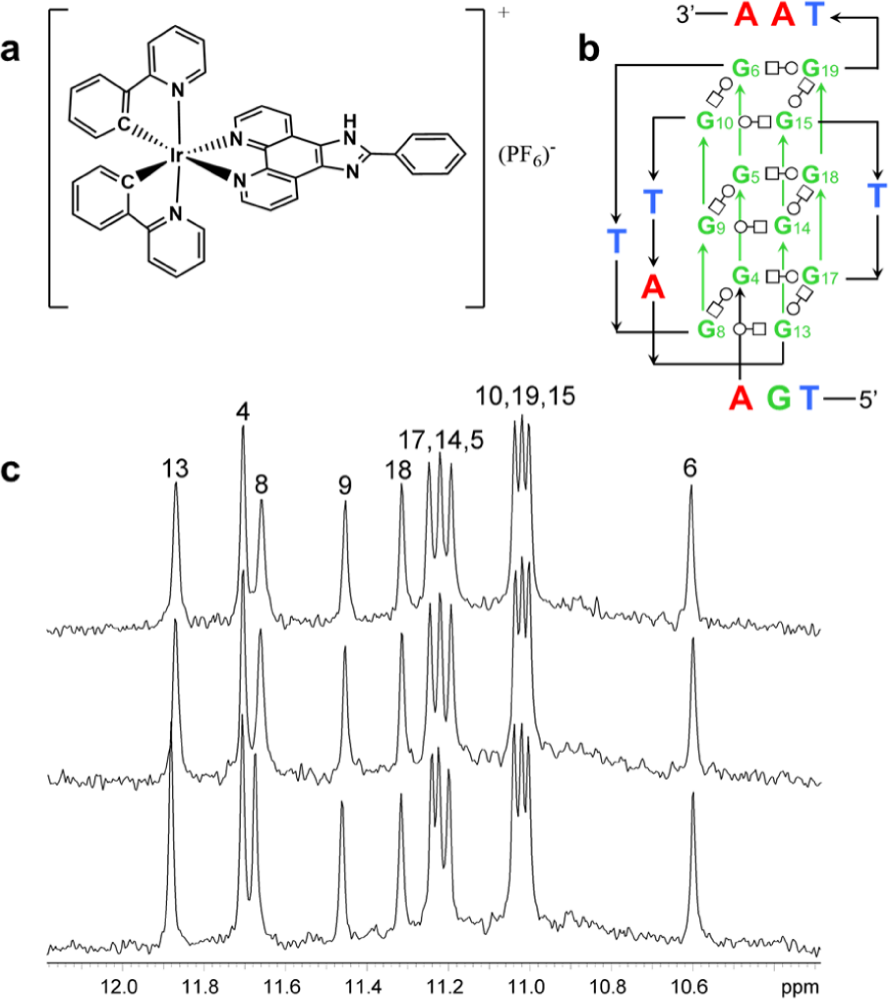
(a) The structure of [Ir(ppy)_2_(pizp)](PF_6_), **1(PF**_**6**_). (b) The secondary structure of **Pu22T** in K^+^ solution. Noncanonical pairing is indicated by Leontis–Westhof symbols.^54^ (c) The imino region of a ^1^H NMR spectra of 0.12 mM **Pu22T** and 3.8% DMSO-d_6_ buffer solution: with no **1(PF**_**6**_) present (bottom); with 0.5 equiv of **1(PF**_**6**_) (middle); with 1.0 equiv of **1(PF**_**6**_) (top). Further additions of **1(PF**_**6**_) beyond one equivalent were limited by the solubility of **1(PF**_**6**_) in the DNA buffer solution. All spectra were acquired with 256 scans at 25 °C and are referenced to DMSO-d_6_ at 2.641 ppm. Imino proton resonances are assigned to the guanine residues as indicated above each peak. See Figure S2 for the imino region of a ^1^H NMR spectra of 0.13 mM **Pu22T** and 20% DMSO-d_6_ buffer solution.

In this study we delve into the binding interactions between compound **1**, [Ir(ppy)_2_(pizp)]^+^, and GQ **Pu22T** (Figure 1). Our aim is 3-fold: first, to assess the binding site through nuclear magnetic resonance (NMR) spectroscopy; second, to evaluate the predictive capabilities of molecular docking in identifying a binding site consistent with experimental data; and third, to explore the refinement possibilities of molecular dynamics in refining the binding interactions estimated by docking.

NMR spectroscopy is highly effective for characterizing ligand–GQ interactions. It allows us to gain insights into the structure and dynamics of the interaction in solutions that mimic physiological conditions.^24−26^ There are many GQ sequences that would be interesting to study as the target of ligand interactions. In this work, we chose **Pu22T**, a mutated sequence of the 27-mer nuclease hypersensitivity element III_1_ sequence of the c-MYC promoter, Pu27. It is an ideal candidate because of the biological significance and because it adopts one major conformation in solution, facilitating the identification of structural changes induced by binding.^27,28^

Molecular docking offers a fast and cost-effective computational method for predicting ligand-macromolecule binding modes. Though developed for the investigation of ligand-protein interactions, it is being increasingly applied to ligand-nucleic acid systems.^29−33^ In our investigation, we utilize NMR data to scrutinize docking output, aiming to determine the efficacy of docking as a screening method for future GQ ligands. Finally, molecular docking is most robust when used in conjunction with molecular dynamics simulations for the design of ligands for GQ biosensors.^34^ Here we investigate the stability of the ligand–GQ binding pose predicted by docking over the course of 1 μs molecular dynamics simulations.

Based on NMR studies, it appears that **1** binds to **Pu22T** through pi stacking below the 5′ G-tetrad, with the strongest interactions occurring with G8 and G13. Molecular docking modeled binding in this region of **Pu22T** only when cross-docking^29^ was conducted, in which we used a structure of **Pu22T** that had been determined with another ligand^35^ and we manually removed the ligand from the structure prior to docking with **1**. Subsequent 1 μs molecular dynamics simulations revealed the stability of the ligand–GQ binding pose predicted by docking. Notably, ligand **1** remained positioned below the 5′ G-tetrad throughout the simulation, repositioning with root-mean-square deviations (RMSDs) of less than 4.5 Å, to adopt a configuration optimizing overlap with G8 and G13.

## 2 Materials and Methods

### 2.1 Materials

1,10-Phenanthroline-5,6-dione (97%), benzaldehyde (99.5%), 2-phenylpyridine (98%) (ppy), iridium(III) chloride hydrate (reagent grade), and acetonitrile-d_3_ (99.8%) were purchased from Sigma-Aldrich. Ultra pure grade ammonium acetate was purchased from Amresco. Glacial acetic acid was purchased from Fisher Scientific. DMSO-d_6_, 99.9% and D_2_O-d_2_, 99.9%, were purchased from Cambridge Isotope Laboratories. Potassium chloride (99.9%), potassium monobasic (99.7%), and potassium dibasic phosphate (99.7%) were purchased from J. T. Baker. All chemicals were used as received.

### 2.2 Synthesis

The iridium dimer, [Ir_2_(ppy)_4_Cl_2_], was prepared according to a previously reported procedure.^36^ 2-Phenylimidazole[4,5f][1,10]phenanthroline (pizp) was prepared using a CEM Mars 6 microwave according to a previously reported procedure.^37^ [Ir(ppy)_2_(pizp)](PF_6_), **1**, was prepared according to a previously reported procedure.^38^ The NMR spectrum of **1** was collected in acetonitrile-d_3_ on a Bruker 300 MHz NMR spectrometer (Figure S1).

### 2.3 DNA Sequences and Sample Preparation

The nucleic acid was purchased from Integrated DNA Technologies with standard desalting completed. The sequence was 5′-TGA GGG TGG GTA GGG TGG GTA A-3′ (**Pu22T**). Buffer exchange was conducted twice using an Amicon Ultra 4 3000 MW cutoff filter and potassium phosphate buffer, with the first exchange containing 0.05 mM EDTA. Buffer was 70 mM potassium chloride, 25 mM potassium phosphate, and pH = 6.9. DNA samples were annealed in an NMR tube by placing tube in a 95 °C water bath for 15 min and then removing the tube from the water bath and allowing it to cool to room temperature. NMR samples were prepared with 96.2% buffer and 3.8% DMSO-d_6_ or D_2_O by volume or 80.0% buffer and 20.0% DMSO-d_6_ by volume. Sample solutions contained 1.4 mM DNA oligonucleotides for peak assignments with 2D NOESY NMR and 0.12–0.13 mM DNA for titration experiments.

### 2.4 NMR Studies

NMR studies involving **Pu22T** and **1(PF**_**6**_) were conducted on a Varian Unity INOVA 600 MHz NMR spectrometer. The NMR solution structure of **Pu22T** was previously characterized,^27^ and this characterization provided initial guidance for peak assignments with 2D NMR. Chemical shifts of guanine imino protons are shown in Table S1. Note that in reference^27^**Pu22T** residues are labeled with numbers from 4 to 25. We have chosen to use a shifted numbering scheme of 1–22 as this corresponds directly to the numbering scheme in the Protein Data Bank for PDB ID: 1XAV. For example, guanine 4 in reference^27^ is guanine 1 here.

For titration experiments in 3.8% DMSO-d_6_, 50 μL of 1.24 mM DNA was diluted with 450 μL buffer and 20 μL DMSO-d_6_ to create a 0.12 mM DNA and 3.8% DMSO-d_6_ solution. A 14.7 mM solution of complex **1(PF**_**6**_) in DMSO-d_6_ was spiked into the DNA in two, 2 μL aliquots, resulting in an approximate 1:1 ratio of complex **1(PF**_**6**_) to DNA. For titration experiments in 20% DMSO-d_6_, 50 μL of 1.24 mM DNA was diluted with 350 μL buffer and 100 μL DMSO-d_6_ to create a 0.13 mM DNA and 20% DMSO-d_6_ solution. A 14.7 mM solution of complex **1(PF**_**6**_) in DMSO-d_6_ was spiked into the DNA in four, 2 μL aliquots, resulting in an approximate 2:1 ratio of complex **1(PF**_**6**_) to DNA. NMR peaks of **Pu22T** complexed with **1(PF**_**6**_) were identified by tracking in the 1D titration spectra. The impact on chemical shifts due to added DMSO with each titration were compensated for by measuring shift changes at 4%, 10% and 20% DMSO with no ligand present (Table S1) and subtracting the linearly interpolated DMSO impact.

### 2.5 Docking Studies

Macromolecules (GQs) and **1** were prepared for docking from crystal structures. The quindoline ligand was prepared for redocking from the crystal structure of PDB ID: 2L7V. Crystal structures of G-quadruplexes (GQs) were obtained from the Protein Data Bank (PDB ID: 1XAV and 2L7V). When multiple models were present in the PDB file, the first model was always used. The crystal structure of **1(PF**_**6**_) was obtained from the Cambridge Crystallographic Database (CCDC ref. number: 628443).^39,40^ The CIF file of **1(PF**_**6**_) was converted to a PDB file in Mercury.^41^ For both macromolecule and ligand, solvents and ions were deleted in ChimeraX.^42^ In the case of 2L7V for cross-docking, quindoline ligands were also deleted in ChimeraX. Gasteiger partial charges were added to both macromolecule and ligand in AutoDock Tools. Charges were manually adjusted on [Ir(ppy)_2_(pizp)]^+^, in the.pdbqt file, to give an overall charge of +1. This was done by assigning Ir a +3 charge and each C and N donor atom on the phenylpyridine ligands an additional −0.5 charge. Charges were then redistributed as in previous work, transferring +0.2 charge from iridium to each of the six donor atoms resulting in a +1.8 charge on the Ir atom (Supporting Information).^43,44^ Ir parameters were added to the atom parameter file as follows: Rii = 2.96 Å, epsii = 0.56 kcal/mol, vol = 55.0585, and solpar = −0.00110, utilizing the volume of iodine and previously published parameters of ruthenium.^45^

Grids were created with 100 points in each dimension. The grid of **1XAV** was centered with at x-center = −0.049, y-center = −11.835, and z-center = 0.970. The grid of **2L7V** was centered with x-center = 0.659, y-center = −0.627, and z-center = −4.749. The Lamarkian genetic algorithm was used to perform rigid docking with 2.0 × 10^9^ generations and either 2.0 × 10^7^ energy evaluations (**1** with **1XAV** or **2L7V**) or 2.0 × 10^9^ energy evaluations (quindoline with **2L7V**).^44^ Docking was carried out 100 times for each GQ-ligand pair, with each run using a new random seed. Docked poses were then clustered based on binding energy with a 2.0 Å energy tolerance in AutoDock Tools. Each cluster was then visually analyzed to ensure all poses occurred in the same region of the molecule.

### 2.6 Creating Parameters and Input Coordinates for MD Simulations

The [Ir(ppy)_2_(pizp)]^+^ frcmod parameter file and RESP charges were generated using the MCPB.py included with AmberTools21.^46,47^ To ensure the MCPB.py program recognized the organometallic Ir–C bonds, the bind atoms line of the gene_model_files.py file was edited to include carbon as follows: BIND_ATOMS = [“C”, “N”, “O”, “S”, “F”, “Cl”, “Br’, “I”]. A +3 charge was added to the IR.mol2 file and a −2 charge was added to the RES.mol2 file prior to running MCPB.py calculations (Supporting Information). The lowest energy docked GQ/[Ir(ppy)_2_(pizp)]^+^ pose generated by Autodock 4.2 was used as the starting coordinates for MD simulations. Based on Havrila et al., tleap was used to generate the topology and input coordinate files for the MD simulations using the following parameters: OL15 and gaff force fields, Joung and Cheatham ion with SPC/E water models, and a truncated octahedron water box of 11 Å.^48−50^ Potassium ions were added to neutralize the DNA charge. Based on the volume of the water box, K^+^ and Cl^–^ ions were then added to obtain a 0.2 M KCl concentration.^50^

### 2.7 Molecular Dynamics Simulations

All systems were prepared for molecular dynamics productions through a series of 13 minimization (min), heating (heat), and equilibration (equil) steps as follows: min, heat, min, equil, min, equil, min, equil, min, equil, min, equil, and equil. In systems containing only nucleic acids, all residues were restrained. In ligand/nucleic acid systems, the ligand and first 20 residues of the nucleic acid were restrained. Restraints to the solute atoms during minimizations, heating, and equilibrations were slowly relaxed as in Havrila et al.^50^ A 9.0 Å nonbonded cutoff was used for all minimizations, heating, equilibrations, and productions.

Energy minimizations consisted of 500 steepest descent steps followed by 500 conjugate gradient steps. After the first minimization, the system was heated from 5.0 to 298.15 K over 50 ps using a Langevin thermostat with 5 ps^–1^ frequency of collision. SHAKE constraints were applied to all hydrogens during heating. Equilibrations were carried out for 50 ps at 298.15 K and 1 atm with Monte Carlo pressure regulation and isotropic position scaling turned on. 100 steps were used between volume change attempts. SHAKE constraints were applied to all hydrogens during equilibration.^51^

From the 13th equilibration step output, four 50 ps equilibrations with no atomic restraints were conducted, each with a unique random seed based on the clock. Finally, production simulations were run, each with a unique random seed based on the clock, at 298.15 K and 1 atm (NPT ensemble), hydrogen bonds constrained with SHAKE algorithm, and a 2 fs time step was applied. Each quadruplex or ligand/quadruplex complex was simulated in four independent productions for 1.2 μs each. All analyses were conducted on the last 1 μs of the simulations (from time 0.2–1.2 μs).

### 2.8 Analysis of MD Simulations

Mass-weighted root-mean-square deviation (RMSD) of nucleic acids throughout the trajectory compared to input structure and distances between atoms throughout the trajectory were calculated using the cpptraj module of Amber20.^47^ Final ligand positions from **2L7V-1** MD simulations were compared to the input ligand position by aligning nucleotides G4, G8, G13, and G17 of both structures in Pymol using the align command.^52^ The RMSDs of ligand (**1**) in the final MD poses of **2L7V-1** compared to the input position were calculated using the RMSD command in ChimeraX.42

## 3 Results and Discussion

### 3.1 NMR Binding Studies

NMR spectroscopy is an effective method to structurally determine GQ-ligand binding interactions. **Pu22T** lends itself well to this technique as it exists primarily in a single topology in solution and resonances have already been assigned.^27^ The interactions between **1(PF**_**6**_) and **Pu22T**, were investigated with 1D ^1^H NMR titrations in 3.8% and 20% DMSO-d_6_ buffer solutions (Figures 1 and S2). We focus our discussion on results from the 3.8% DMSO-d_6_ solutions as these resulted in the smallest disruption of the GQ NMR resonances compared to pure buffer (Table S1). Results from the 20% DMSO-d_6_ solutions, which allowed higher ligand:GQ ratios, are provided in Supporting Information (Figures S2 and S3) and support conclusions. Titrations followed the changes in proton chemical shifts as **1(PF**_**6**_) was added to an NMR tube containing annealed **Pu22T**. In the ^1^H NMR titration spectra (Figure 1c), the number of imino peaks remain constant and no new peaks appear, indicating the binding process occurs at a rapid exchange rate relative to the NMR time-scale. The unchanged number of imino peaks and rapid exchange rate are consistent with binding results observed by Calabrese et al. when titrating DC-34 with **Pu22T**.^53^

**Figure 2:**
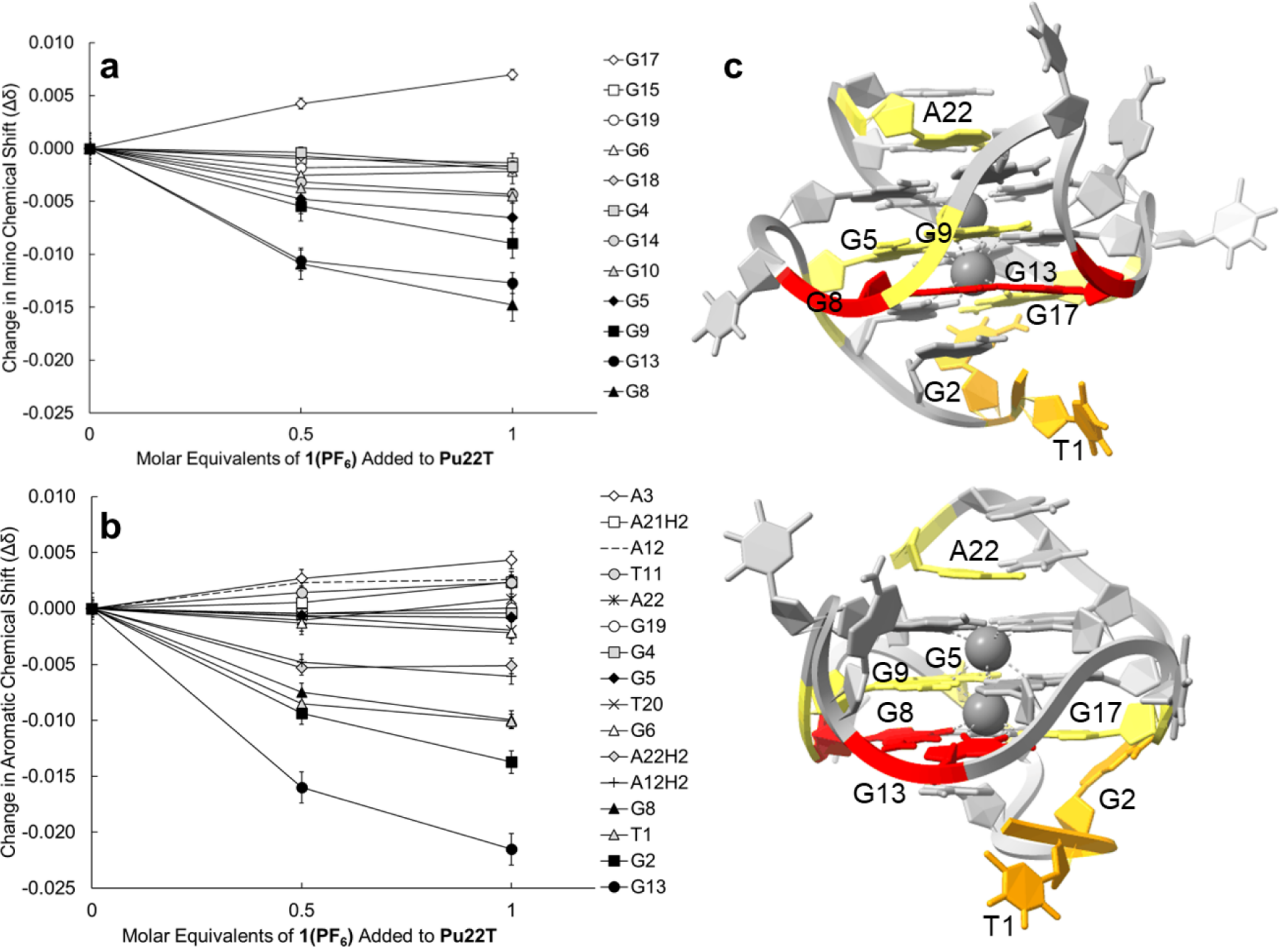
(a) The change in chemical shift of the guanine imino protons as **Pu22T** in a 3.8% DMSO-d_6_ buffer solution was titrated with **1(PF**_**6**_). (b) The change in chemical shift of the aromatic protons as **Pu22T** was titrated with **1(PF**_**6**_). (c) Color visualization of the change in chemical shifts of **Pu22T** (PDB ID: 1XAV) from two different viewpoints. The residues of **Pu22T** that experienced small (0.005–0.009 ppm) changes are shown in yellow, moderate changes (0.010–0.014 ppm, orange) and relatively large changes (>0.014 ppm, red) in the presence of one equivalent of **1(PF**_**6**_). Error bars are the standard deviation of five readings of the **Pu22T** titrated with 0.5 and 1 equiv of **1(PF**_**6**_) in 20% DMSO-d_6_ buffer solution.

The magnitude of changes to **Pu22T** proton chemical shifts as a result of the titration are shown in Figure 2. The largest change to imino proton chemical shifts were observed for the G13 and G8 imino protons (Δδ: −0.0125 and −0.0149 ppm respectively) and moderate changes were observed for G17, G9, and G5 imino protons (Δδ: 0.0071, −0.0089, and −0.0065 ppm respectively). All other imino protons experienced shift changes less than |0.005| ppm in the presence of **1(PF**_**6**_). The changes to the imino chemical shifts of G17, G13, and G8 are consistent with pi stacking of a ligand at the 5′ end, as observed with previously reported bound quindoline, DC-34, and BMVC ligands.^28,53,55^ We do not see significant shifts of any imino protons associated with the 3′ tetrad. This is consistent with the work of Liu et al. where, when only one equivalent of BMVC was present, binding at the 5′ end was preferred.^55^ Additionally, Deng et al. conducted binding free energy calculations, using multiple computational methods, showing binding at the 5′ end to be 1–5 kcal/mol more stable compared to the 3′ end.^56^ In the case of quindoline and DC-34 when pi stacking occurred at both the 5′ tetrad and the 3′ tetrad, changes in imino shifts were observed for G15 along with G6 or G19.^28,53^ While we were not able to titrate with two equivalents of ligand due to solubility limitations in the 3.8% DMSO-d_6_ buffer solution, it is worth noting that Calabrese et al. saw significant movement of G15 and G6 imino chemical shifts even in the presence of only one equivalent of ligand.^53^

In the aromatic spectral region, the largest chemical shift change was noted for G13(H8) (Δδ: −0.0215 ppm). Moderate shifts were observed for G2(H8) (Δδ: −0.0137 ppm), T1(H6) (Δδ: −0.0099 ppm), and G8(H8) (Δδ: −0.0099 ppm). A relatively small shift was observed for A22(H2), (Δδ: −0.0047 ppm). Similar changes to chemical shifts for G13(H8) and T1(H6) were observed in the presence of quindoline and BMVC while the change to G8(H8) was only observed in the presence of BMVC.^28,55^ In the previously reported Pu22T-DC-34 and Pu22T-BMVC structures, T1 and G2 were found to move to stack over each other once the ligand was bound to create a hydrophobic pocket.^53,55^ This may explain the change in chemical shifts we observed for T1 and G2. Similar changes at the 3′ end were previously observed when ligands were bound at both ends, where A21 and A22 were no longer stacking but rather move away from the tetrad to accommodate the ligand.^28,53,55^ However, without changes in the resonances of the 3′ G-tetrad, in combination with the small change observed for A22, it is hard to conclude that binding is also occurring at the 3′ end. These findings led us to hypothesize the following potential binding scenarios: intercalation between the 5′ (G8, G13, G17) and middle tetrad (G5, G9) or binding via end-stacking at the 5′ tetrad. Eight solution structures of ligand-Pu22 or ligand-Pu22T are known and in all cases the ligands end-stack above the 3′ and below the 5′ tetrads.^21^ Additionally, it is worth noting that if binding is only occurring at one tetrad, we would expect preference at the 5′ end based on binding free energy calculations conducted by Deng et al. and previous ligand binding studies.^55,56^ Therefore, based on our NMR data, we believe it is likely that **1** is binding via pi-stacking at the 5′ tetrad of **Pu22T**.

### 3.2 Docking Studies

We used molecular docking to assess the efficacy of docking in predicting poses consistent with experimental NMR data. In choosing a docking software, the following criteria were considered: (1) ability to dock small molecules with nucleic acids; (2) ability to incorporate transition metal atoms; and (3) free and open-source availability. While many molecular docking programs have been developed for docking with proteins, some have been developed specifically for nucleic acids. Of those that were developed for proteins, many have been tested for their accuracy with nucleic acids.^29,44,57^ In one study, Dock 6.0 was shown to be the most accurate free, open-source docking software for G-quadruplex nucleic acids, however, it currently cannot incorporate transition metal complexes.^57^ Autodock 4.2 was ultimately chosen due to its ability to accept the manual incorporation of heavy metal atom parameters while still having a 60–68% success rate of redocking within 2.5 Å in RNA and DNA-ligand complexes.^29,44^ Since this work was completed, the MetalDock tool has been developed.^58^ MetalDock is specifically designed for the docking of transition metal complexes and integrates into AutoDock docking engine, however, is limited to 12 metal atom types, not including iridium.^58^ As the MetalDock parameter library expands, this may become the best choice for open-source, transition metal, nucleic acid docking. Our docking protocol was first tested by blind redocking of the quindoline ligand bound below the 5′ tetrad of 2L7 V, which resulted in 15 clusters. The fifth and sixth lowest energy clusters showed ligand poses stacked below the 5′ tetrad (Figure S4).^35^ Blind docking was then conducted between [Ir(ppy)_2_(pizp)]^+^, **1**, and solution structures of **Pu22T** (PDB: 1XAV and 2L7V).^35,59^ The latter represents a solution structure of Pu22T with bound quindoline ligands, where the quindoline ligands were intentionally deleted for the cross-docking analysis.

**Figure 3:**
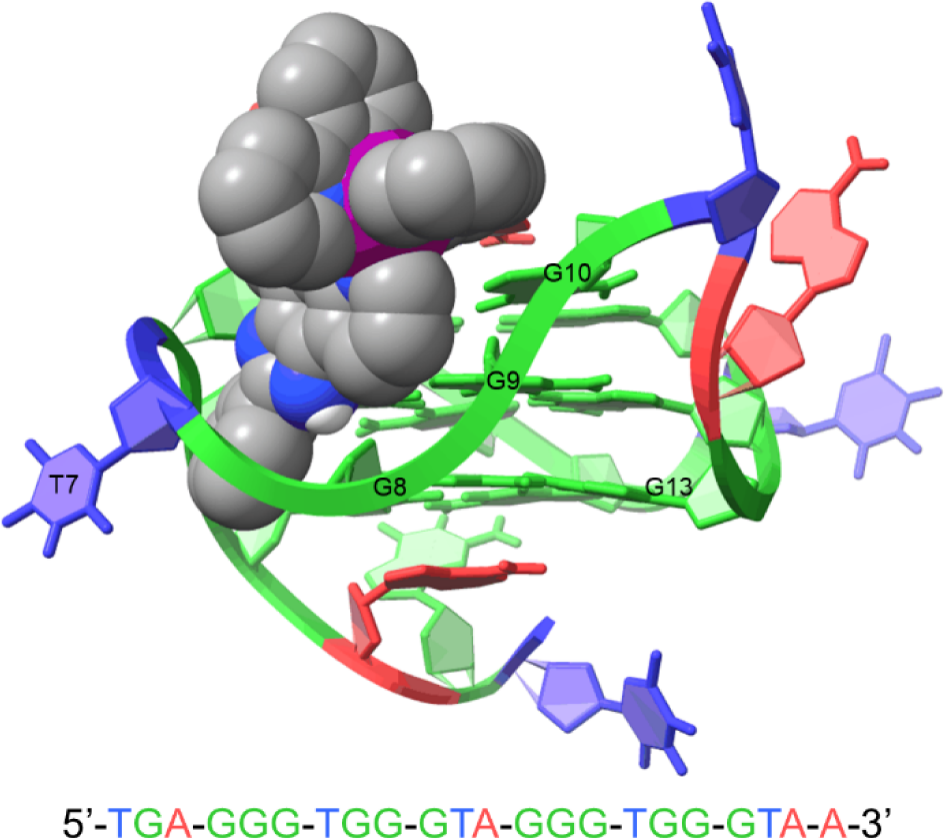
Lowest energy binding pose predicted by Autodock 4.2 for **1** when blind docked with **1XAV**.

Blind docking of **1** over the entire macromolecule, **1XAV**, was repeated 100 times. The clustering (2 Å RMSD) of the 100 output poses led to eight unique clusters with binding energies ranging from −8.87 to −6.97 kcal/mol. Figure 3 shows the lowest energy pose in the cluster with the lowest mean energy. The lowest mean energy cluster contained 51 of the 100 poses and positioned **1** in a loop created by residues G6, T7 and G8. The remaining 7 clusters contain poses where **1** is interacting with a loop or groove of the GQ; there are no interactions with the 3′ or 5′ tetrad of **1XAV** (Figure S5).

**Figure 4:**
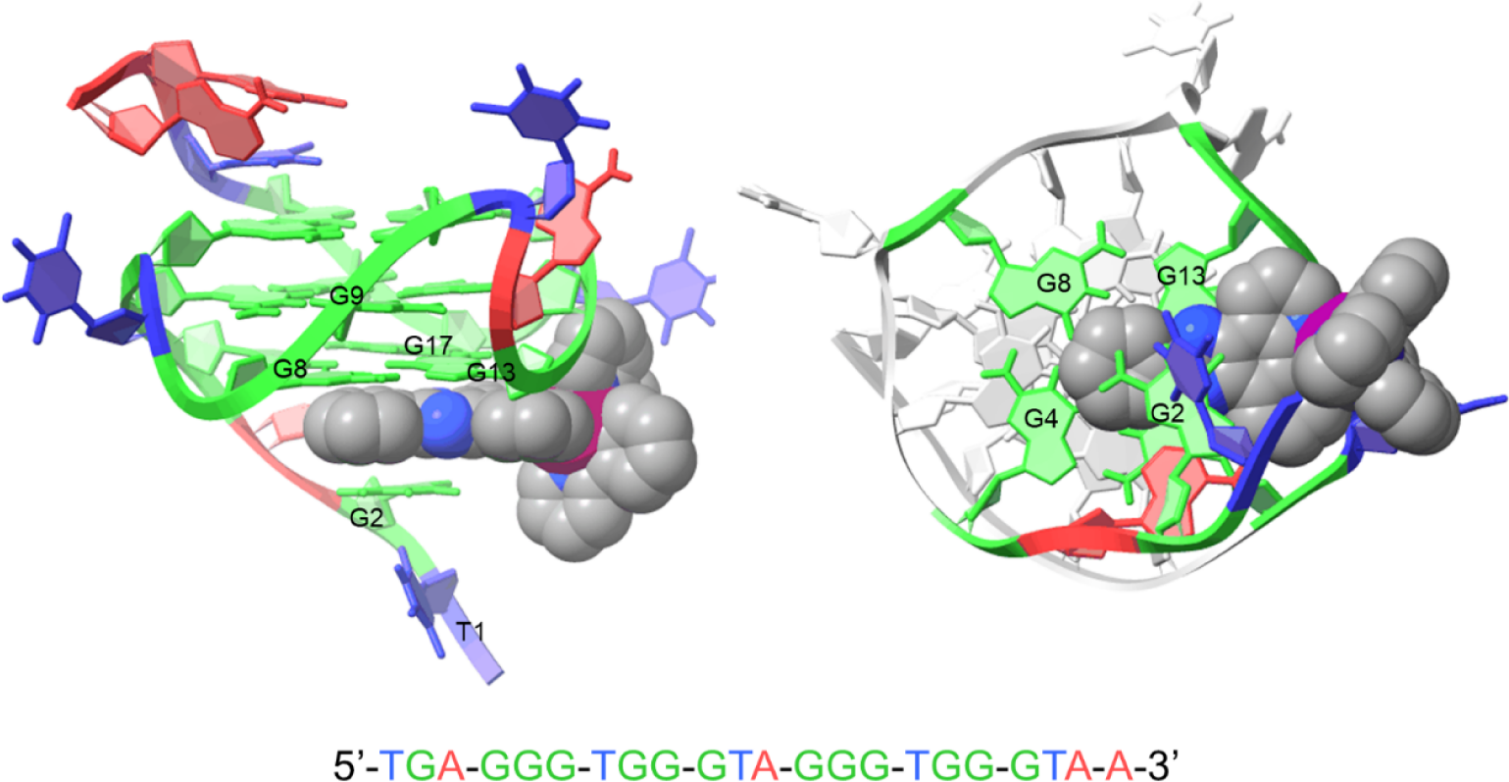
Lowest energy binding pose predicted by Autodock 4.2 for **1** when cross-docked with **2L7V**.

Blind cross-docking of **1** over the entire macromolecule, **2L7V**, was conducted after the quindoline ligands were deleted. The clustering (2 Å RMSD) of the 100 output poses led to eight unique clusters with binding energies ranging from −8.29 to −6.80 kcal/mol. Figure 4 shows the lowest energy pose in the cluster with lowest mean energy. The lowest energy cluster contained 38 of the 100 poses and **1** was centered under residues G13 and G17 at the 5′ end of **2L7V**. This is similar to the pose obtained by Liao et al. when docking a brominated derivative of the pizp ligand with a G-quadruplex.^60^ The remaining clusters include poses of **1** in loops or grooves of the GQ (Figure S6), except for clusters 7 and 8, which show some interaction of phenyl or phenylpyridine rings with residues G6 and G10 (Figure S7). The molecular docking results from the cross-docking of **1** with **2L7V** yielded a lowest energy pose that was consistent with the experimental NMR data. This pose further substantiates our hypothesis that the binding occurs via end-stacking rather than intercalation. In contrast, we attribute the inconsistency between the docking output and NMR data in the **1XAV-1** system to the limited flexibility of the macromolecule in AutoDock 4.2. Specifically, residues A3 and A22 were identified as obstructing the approach of **1** to the tetrads, contributing to the inability of docking to position **1** into a pose that is consistent with the experimental NMR observations.

### 3.3 Molecular Dynamics Simulations

**Figure 5:**
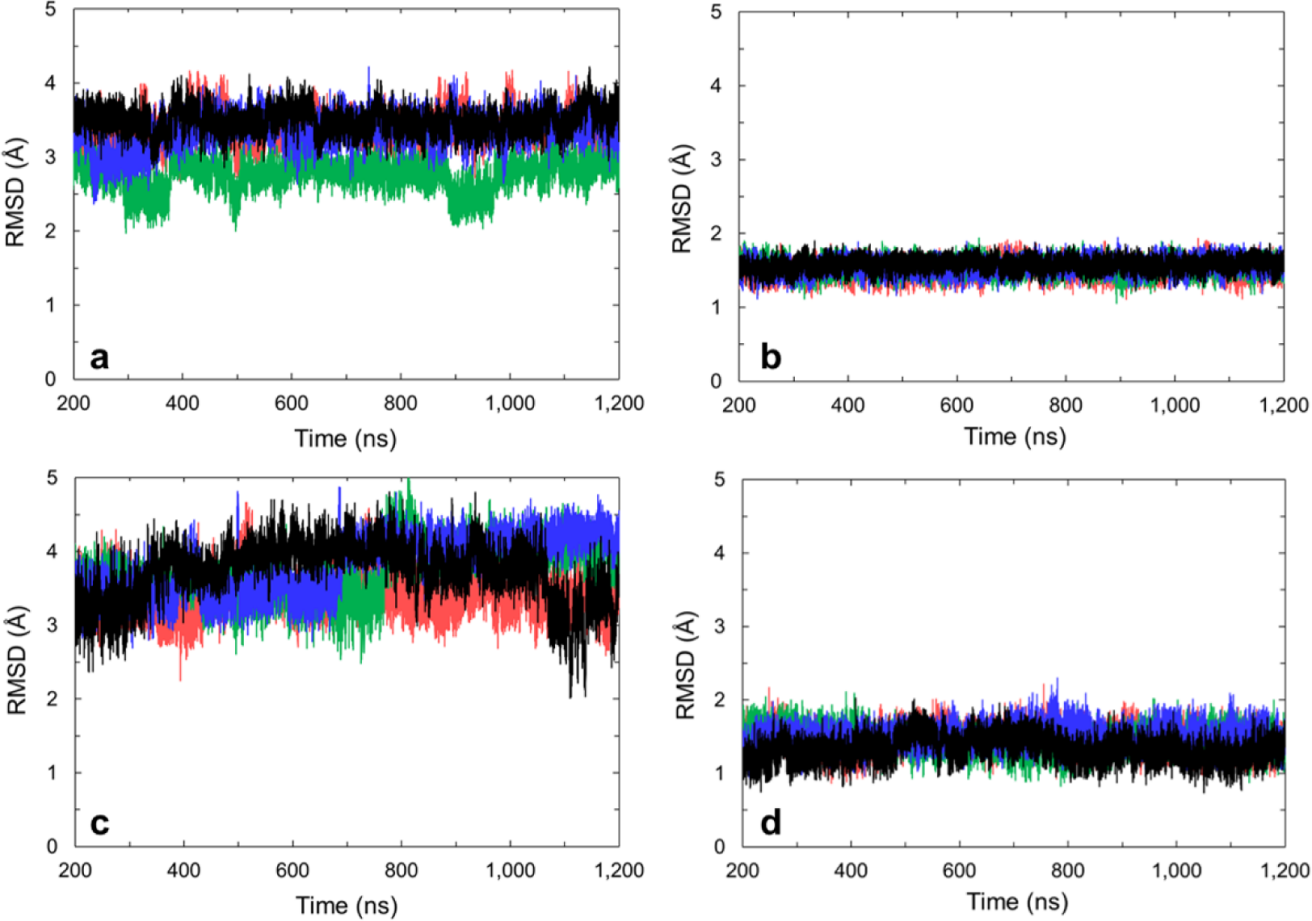
Mass-weighted RMSD to starting structure of (a) all residues in **1XAV** (average 3.2 ± 0.4 Å), (b) only tetrad guanines (4–6, 8–10, 13–15, 17–19) of **1XAV** (average 1.55 ± 0.09 Å), (c) all residues in **2L7V** (average 3.7 ± 0.4 Å), and (d) only tetrad guanines (4–6, 8–10, 13–15, 17–19) of **2L7V** (average 1.4 ± 0.2 Å). Red, black, blue, and green represent four independent simulations.

To determine if **1** was stable in the Autodock predicted poses, molecular dynamics (MD) simulations were performed. Our simulations utilized Joung and Cheatham ion parameters optimized for the SPC/E water model as G-quadruplexes have been shown to be stable for up to five microseconds under these conditions with no channel to bulk ion exchange.^50^ One microsecond long simulations were performed for the free GQs as well as the **GQ-1** complexes. The free GQs, without **1** present, were stable for 1 μs with average RMSDs to the starting structure of ∼3–4 Å (Figure 5). When loops and tails were excluded, the G-tetrads were stable for 1 μs with RMSDs to the starting structure of ∼1.5 Å (Figure 5).

**Figure 6:**
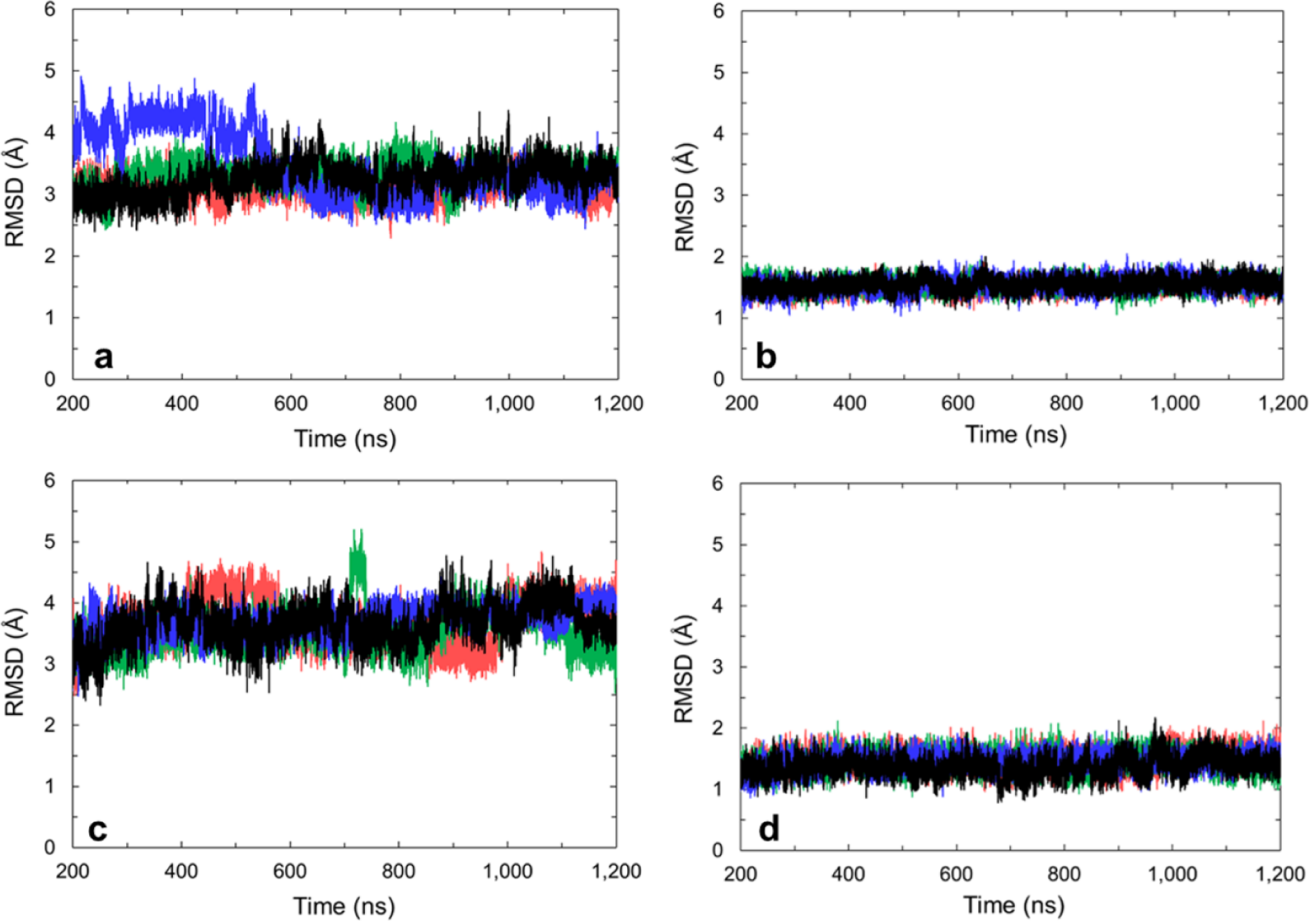
Mass-weighted RMSD to starting structure of (a) all nucleic residues in **1XAV-1** complex (average 3.3 ± 0.3 Å), (b) only tetrad guanines (4–6, 8–10, 13–15, 17–19) in **1XAV-1** complex (average 1.53 ± 0.09 Å), (c) all residues in **2L7V-1** complex (average 3.6 ± 0.3 Å), and (d) only tetrad guanines (4–6, 8–10, 13–15, 17–19) in **2L7V-1** complex (average 1.4 ± 0.1 Å). Red, black, blue, and green represent four independent simulations.

The lowest energy pose from the molecular docking results was used to provide starting coordinates for the GQ-**1** complex MD simulations. When **1** was present, the GQs continued to be stable for 1 μs with average RMSDs of nucleic acid residues to the starting structure of ∼3–4 Å (Figure 6). When loops and tails were excluded, the G-tetrads were stable for 1 μs with an RMSD to the starting structure of ∼1.5 Å (Figure 6).

**Figure 7:**
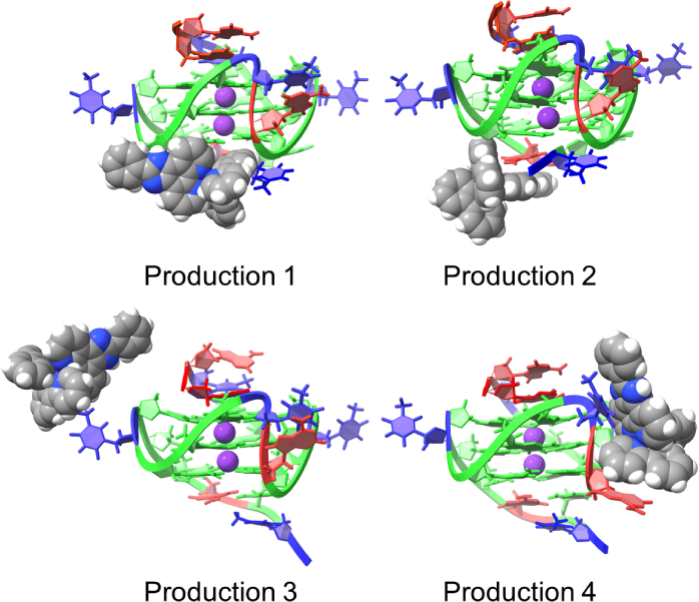
Final positions of **1** when interacting with **1XAV** after 1 μs of MD simulation in each production.

When the GQ was **1XAV**, **1** demonstrated significant movement from the starting position with inconsistent final positions (Figure 7). This demonstrates that the pose of **1** docked to 1XAV was unstable. In the absence of the NMR data that suggested the pose is incorrect, these MD simulations would have also been able to suggest the pose is incorrect.

**Figure 8:**
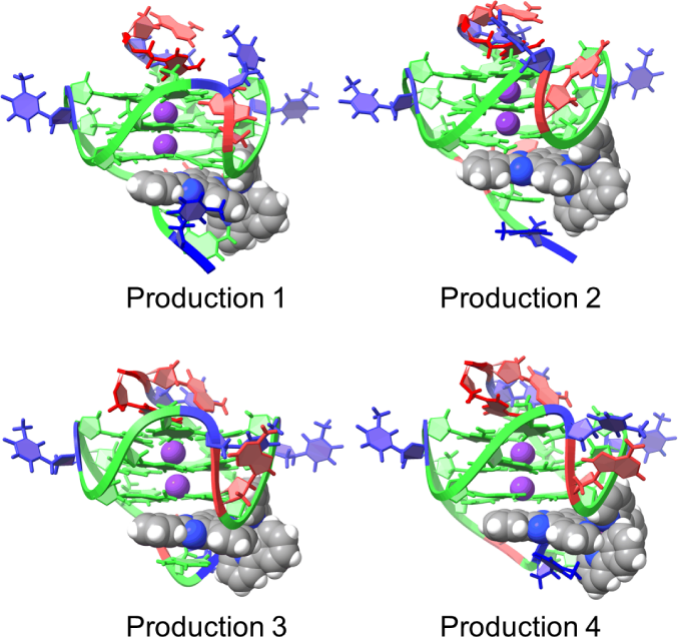
Final positions of **1** when interacting with **2L7V** after 1 μs of MD simulation in each production.

When the GQ was **2L7V**, complex **1** remained under the 5′ tetrad in all productions (Figure 8). The limited movement of the final poses of **2L7V-1** to the large movements noted in **1XAV-1**, reinforces that cross-docking is necessary, in the absence of flexible receptor docking, to enable the ligand to find a stable binding position.

**Figure 9:**
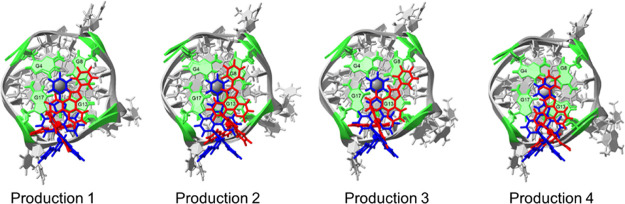
Initial input (blue) and final (red) positions of **1** with **2L7V** from four productions of MD simulations. The initial three nucleotides of the sequence are omitted in this image, and the 5′ G-tetrad is highlighted in green for enhanced visibility. RMSDs between initial and final ligand positions are 3.923 Å, 3.959 Å, 4.243 Å, and 2.608 Å.

Figure 9 shows the change in the position of **1** from the MD input structure of **2L7V-1** to the final frame of each simulation. In all simulations, the pizp ligand of complex **1** moved from lying between the G4/G17 and G8/G13 bases toward the G8 and G13 bases. This movement makes sense as it would increase pi-stacking between the ligand and GQ. To quantify the movement of complex **1** from docking output through MD productions, we monitored the distance between C36 on the ligand molecule and N2 on the G8 (Figure S8). It is interesting to note that the majority of this movement occurred during the minimization and equilibration steps as restraints were relaxed prior to production simulations (Table S2 and Figure S9). We also monitored the distance between ligand C36 and G8 N2 throughout the full productions (Figure S10), where we observe that the ligand largely remains in the binding site and when small excursions occur, it quickly returns (Figure S10). The MD output of **2L7V-1** is consistent with NMR and docking data, indicating stable binding at the 5′ end of **Pu22T**. The MD output is more closely aligned with NMR titration data, particularly for G8 and G13, which exhibited the largest changes in chemical shift (Figure 2). This suggests that MD simulations effectively refine docking poses for ligand-DNA interactions.

## 4 Conclusions

In conclusion, the binding position of [Ir(ppy)_2_(pizp)]^+^, **1**, on **Pu22T** was determined using NMR titrations to occur near residues G5, G8, G9, G13, and G17, indicating an end-stacking interaction at the 5′ end. Blind cross-docking studies and AMBER molecular dynamics simulations corroborated the 5′ tetrad as the lowest energy binding position, with the MD simulation results more closely aligning with the experimental NMR data. In the case of **2L7V-1**, both molecular docking and molecular dynamics simulations successfully predicted the experimentally determined binding position. However, with **1XAV-1**, the rigidity of the macromolecule prevented the exploration of tetrad interactions. If a suitable GQ structure is available for docking, it appears that docking with AutoDock and refinement by Amber MD simulation can successfully model the interactions of iridium(III) complexes with GQs. As we move forward, other GQ-iridium(III) systems will be explored to determine if these methods can be applied more broadly. Locating the **Pu22T-1** binding mode and creating a method for modeling the binding position has implications for the rational design and virtual screening of iridium(III) complexes for GQ biosensors.

## Supporting Information Available

^1^H NMR spectrum of **1(PF**_**6**_**)**; guanine residue imino resonance assignments in **Pu22T**; NMR titration data in 20% DMSO-d_6_ buffer solution; lowest energy poses for all docking clusters; distance monitoring from C36 on ligand to N2 on G8; visualization of ligand position after each minimzation, heating, or equilibration step (PDF)
irpizplig.pdbqt (TXT)
IR.mol2 (TXT)
RES.mol2 (TXT)
