# Supplementary material for: Modeling and NMR Data Elucidate the Structure of a G-Quadruplex–Ligand Interaction for a Pu22T-Cyclometalated Iridium(III) System
Source: J Phys Chem B. 2024 Nov 19;128(47):11634–43. doi: 10.1021/acs.jpcb.4c06262 (PMC11613442; doi:10.1021/acs.jpcb.4c06262)
Supplement: Supplementary file 1 — jp4c06262_si_001.pdf [file jp4c06262_si_001.pdf]

Supporting Information

Modeling and NMR Data Elucidate the Structure of a G-Quadruplex-Ligand Interaction for a Pu22T-Cyclometallated Iridium (III) System

Carly R. Reed[a], Scott D. Kennedy[b], Rachel H. Horowitz[a], Anees Mohammed Keedakkatt Puthenpeedikakkal[b], Harry A. Stern[c], David H. Mathews[b]*

[a]Department of Chemistry and Biochemistry, SUNY Brockport, Brockport, NY 14420, USA, [b]Department of Biochemistry & Biophysics and Center for RNA Biology, University of Rochester Medical Center, Rochester, NY 14642, USA, [c]Orogen Therapeutics, 12 Gill Street Suite 4200, Woburn, MA 01801, USA.

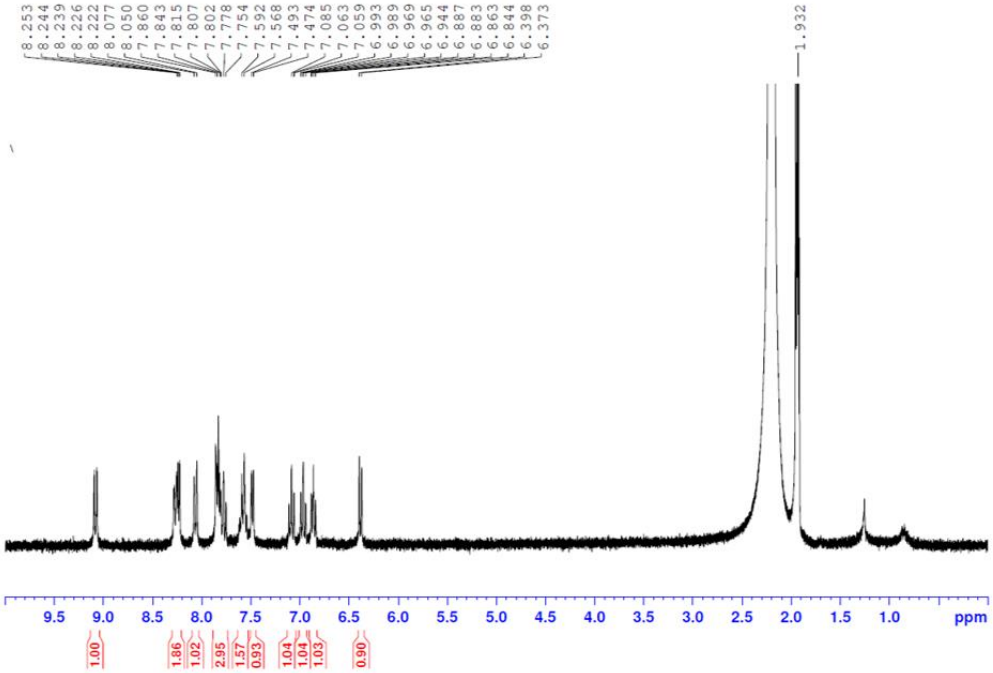

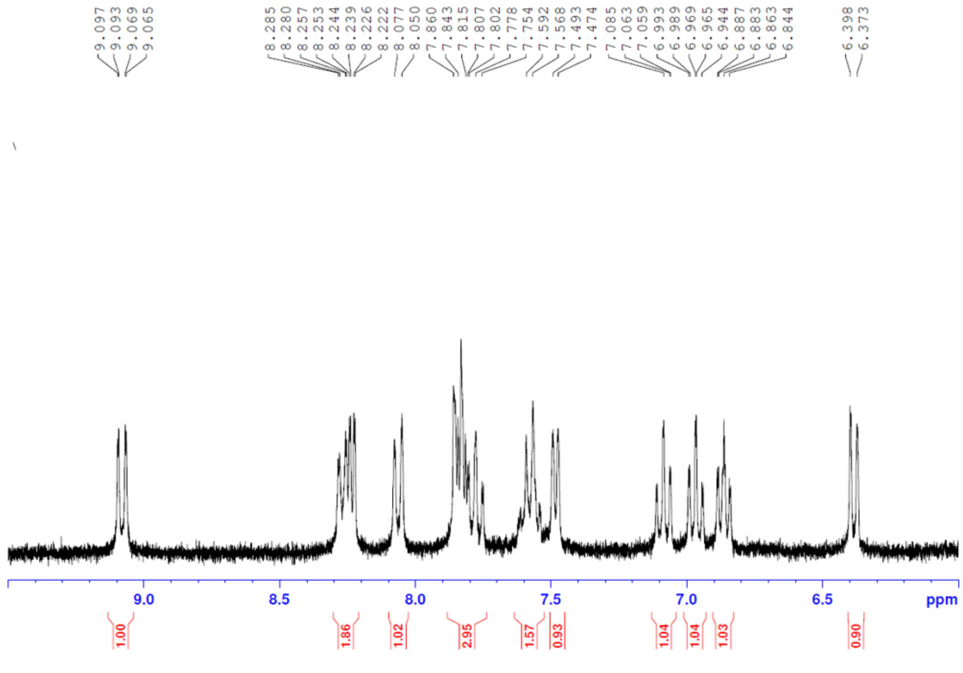

Figure S1. 1H NMR Spectrum of complex 1(PF_6) in acetonitrile-d3. (top) full spectrum, 0-10 ppm; (bottom) zoomed in on aromatic region, 6-9.5 ppm.

Table S1. Guanine residue imino resonance assignments in **Pu22T**.

	Buffer[a]	3.8% DMSO-d6[b]	10% DMSO-d6[b]	20% DMSO-d6[b]
G2				
G4	11.74	11.701	11.698	11.697
G5	11.23	11.194	11.204	11.227
G6	10.61	10.595	10.645	10.751
G8	11.70	11.670	11.677	11.686
G9	11.49	11.457	11.463	11.478
G10	11.06	11.034	11.057	11.110
G13	11.90	11.877	11.896	11.924
G14	11.25	11.219	11.228	11.247
G15	11.03	10.999	11.004	11.017
G17	11.27	11.237	11.239	11.247
G18	11.35	11.312	11.312	11.321
G19	11.06	11.016	11.026	11.051

[a] Chemical shifts referenced to water at 4.75 ppm.

[b] Chemical shifts referenced to DMSO at 2.641 ppm.

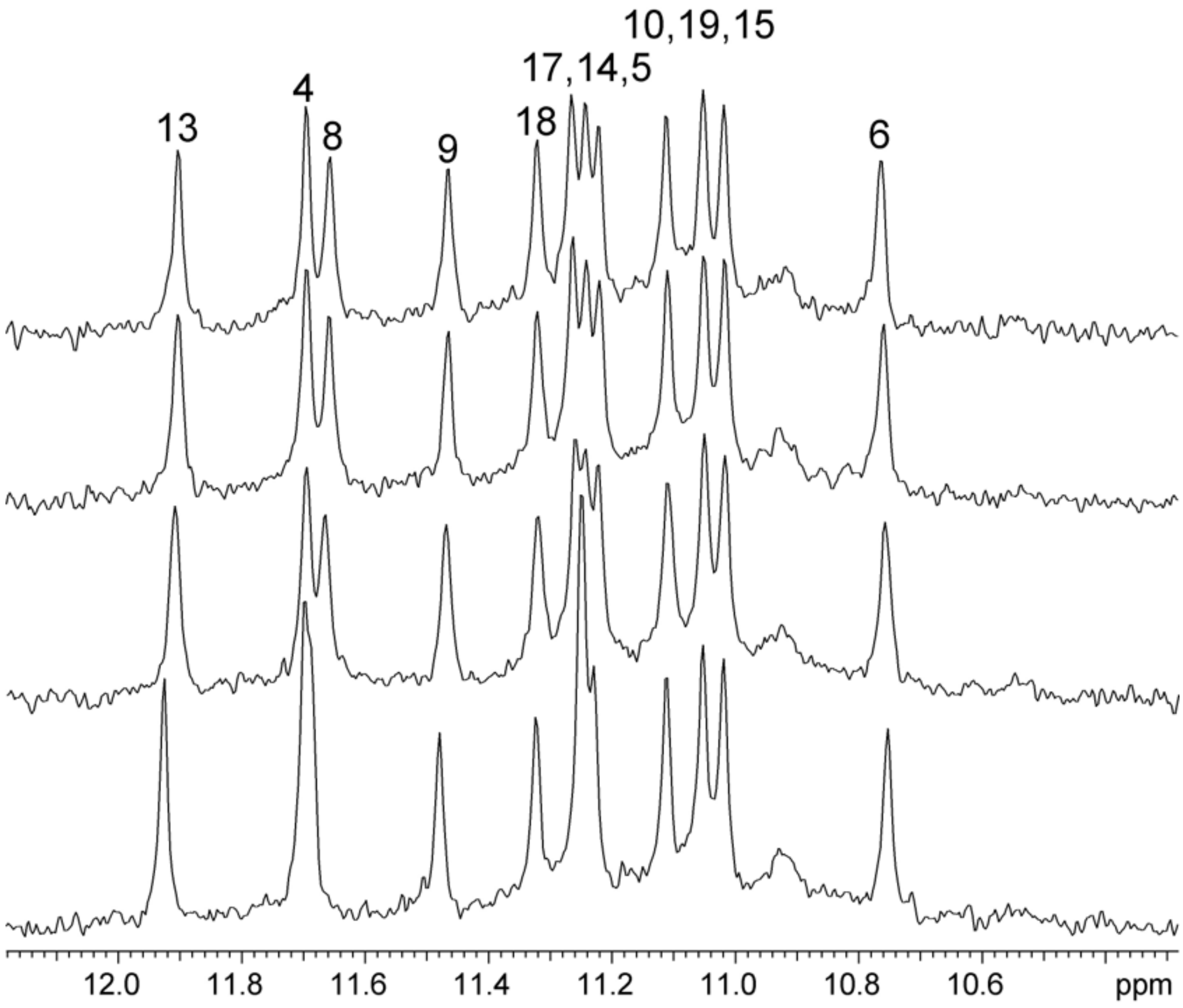

Figure S2. The imino region of a ^{1}H NMR spectra of 0.13 mM Pu22T and 20% DMSO-d6 buffer solution: with no 1(PF_6) present (bottom); with 0.5 equivalents of 1(PF_6) (second from bottom); with 1.0 equivalent of 1(PF_6) (third from bottom); and with 2.0 equivalent of 1(PF_6) (top). All spectra were acquired with 256 scans at 25°C and are referenced to DMSO-d6 at 2.641 ppm. Imino proton resonances are assigned to the guanine residues as indicated above each signal.

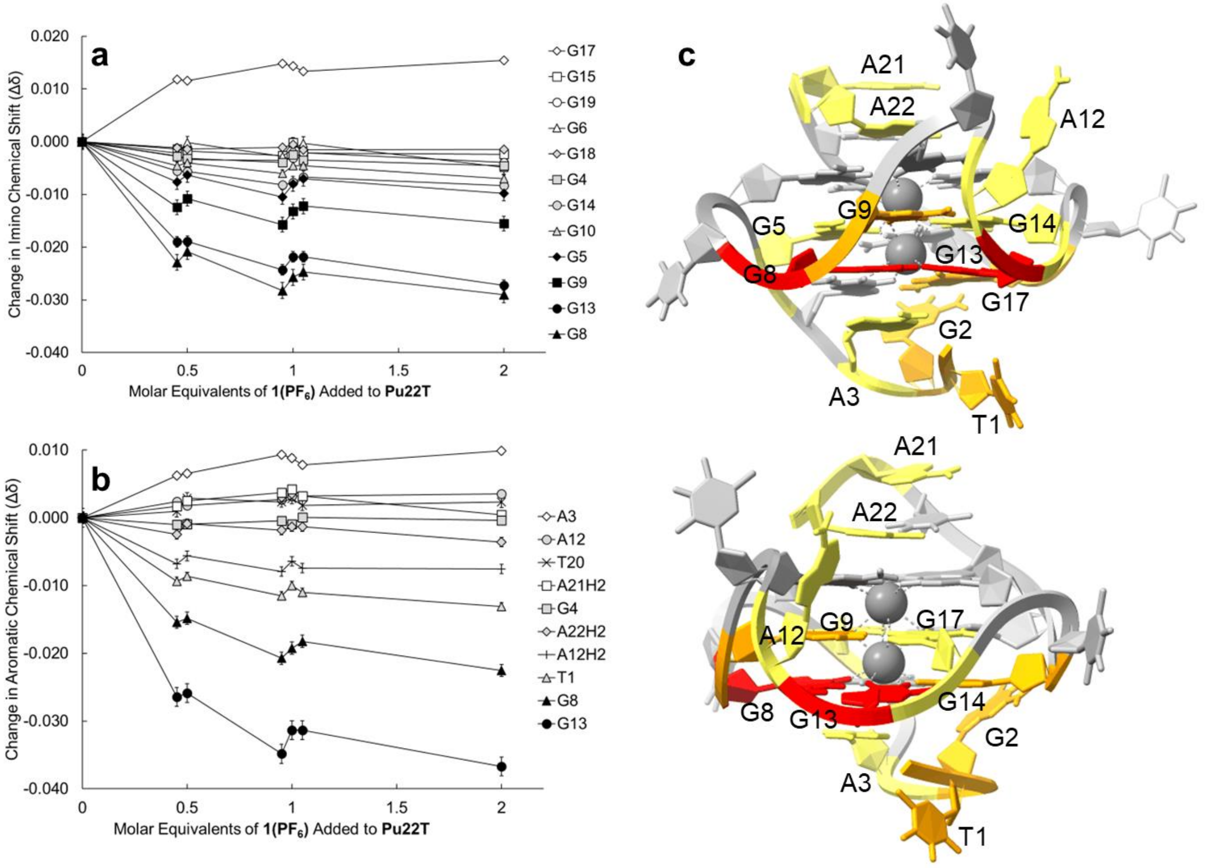

Figure S3. a. The change in chemical shift of the guanine imino protons as Pu22T in a 20% DMSO-d6 buffer solution was titrated with 1(PF_6). b. The change in chemical shift of the aromatic protons as Pu22T in a 20% DMSO-d6 buffer solution was titrated with 1(PF_6). c. Color visualization of the change in chemical shifts of Pu22T (PDBID: 1XAV) from two different viewpoints. The residues of Pu22T that experienced small (0.005-0.009 ppm) changes are shown in yellow, moderate changes (0.010-0.014 ppm, orange) and relatively large changes (>0.014 ppm, red) in the presence of one equivalent of 1(PF_6). Error bars were derived from the standard deviation of five readings of Pu22T titrated with 0.5 and 1 equivalent of 1(PF_6).

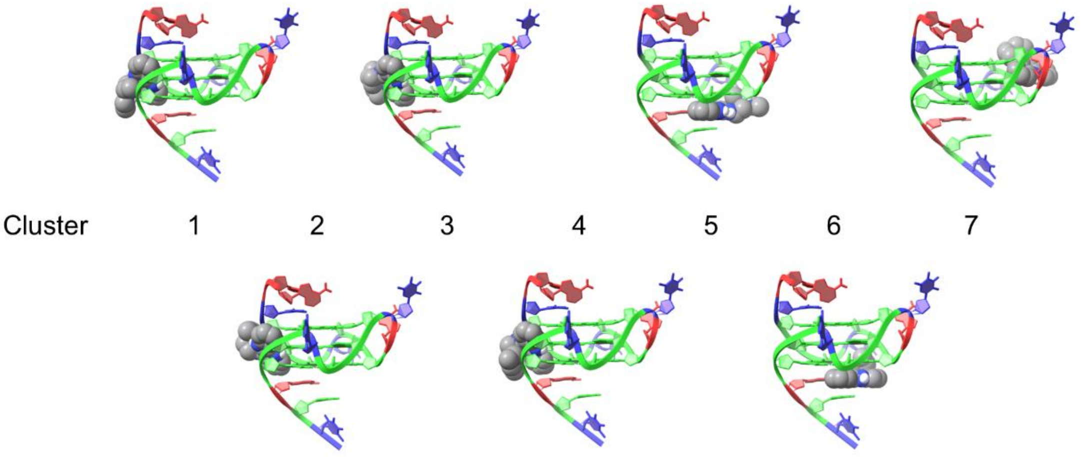

Figure S4. Lowest energy binding pose from each cluster 1-7 predicted by Autodock 4.2 when blind re-docking the 5’ quindoline ligand was conducted with 2L7V. The RMSD of each clusters lowest energy pose compared to crystal structure position are: (1) 18.058 Å, (2) 19.017 Å, (3) 18.485 Å, (4) 17.736 Å, (5) 7.276 Å, (6) 9.150 Å, (7) 13.952 Å.

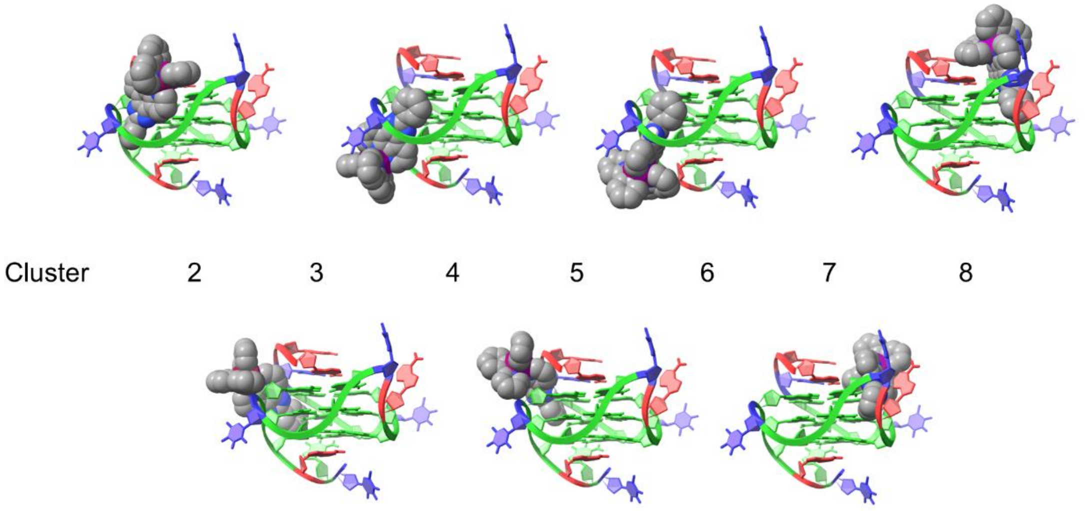

Figure S5. Lowest energy binding pose from each cluster 2-8 predicted by Autodock 4.2 when blind docking 1 with 1XAV.

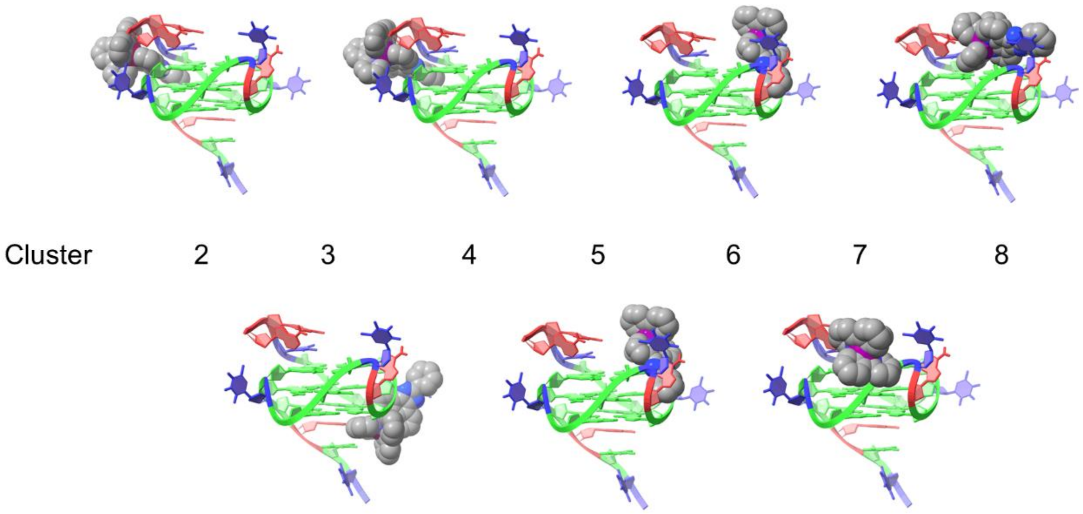

Figure S6. Lowest energy binding pose from each cluster 2-8 predicted by Autodock 4.2 when cross docking 1 with 2L7V.

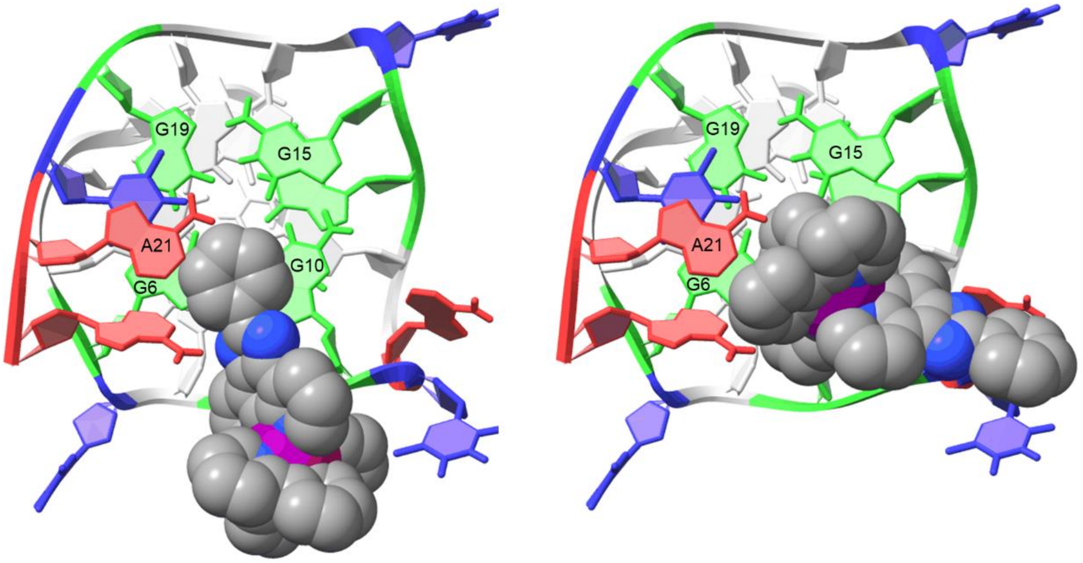

Figure S7. Lowest energy binding pose from cluster 7 (left) and 8 (right) predicted by Autodock 4.2 when cross-docking 1 with 2L7V.

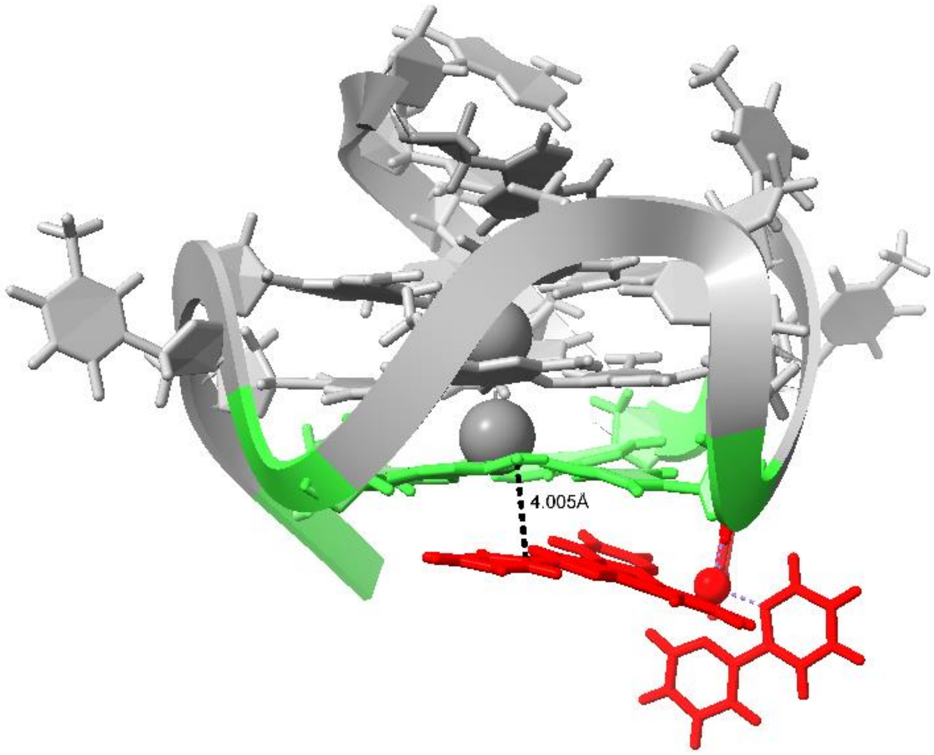

Figure S8. The distance monitored from C36 on ligand to N2 on G8, shown here for production 1.

Table S2. Distance between ligand C36 and G8 N2 atoms at the completion of each step.

Simulation Step	Restraints $kcal \cdot mol^{-1} \cdot Å^{-2}$	Distance from C36 to N2 (Å)
Min 1	25	6.638
Heat 2	25	6.562
Min 3	5	6.540
Equil 4	5	6.320
Min 5	4	6.292
Equil 6	4	6.020
Min 7	3	6.050
Equil 8	3	6.048
Min 9	2	5.916
Equil 10	2	5.654
Min 11	1	5.791
Equil 12	1	5.583
Equil 13	0.5	5.219
Equil 14 (prod 1)	0	4.381
Production 1	0	4.005

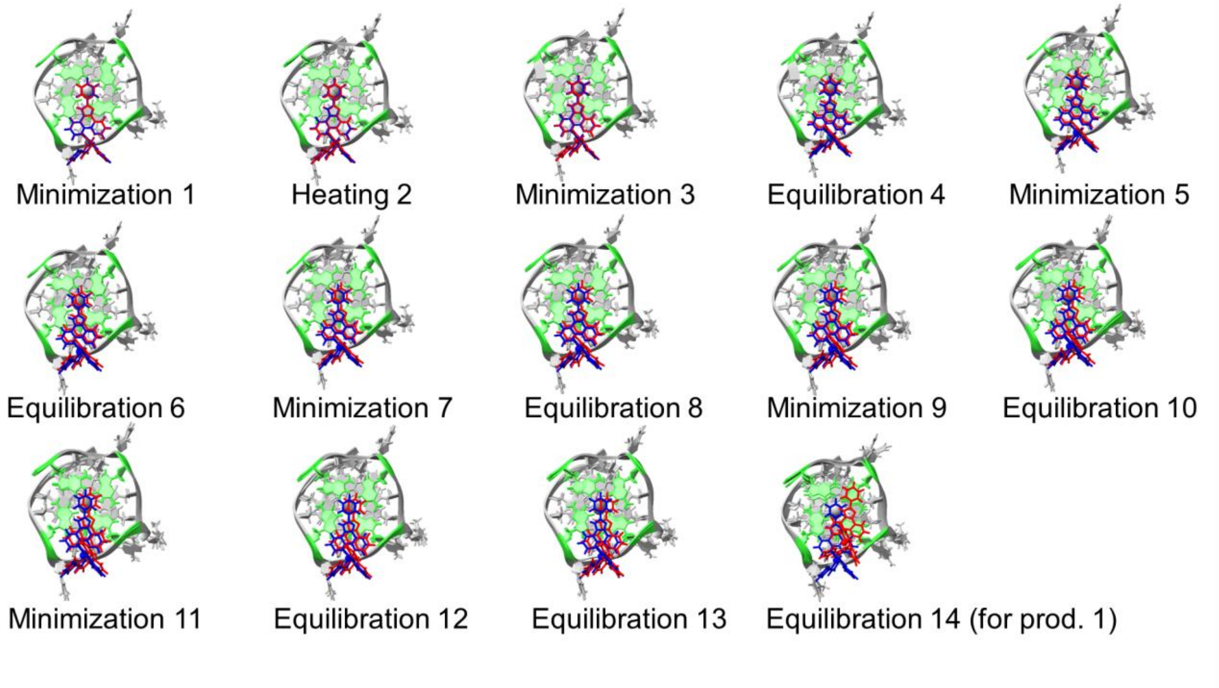

Figure S9. Visualization from below tetrad of ligand position (blue) from each minimization, heating, or equilibration step run prior to full productions. Equilibration step 14 is shown for production simulation one, but was conducted for each of the four production simulations. Docking output ligand position is shown in red. The initial three nucleotides of the sequence are omitted in this image and the 5' G-tetrad is highlighted in green for enhanced visibility.

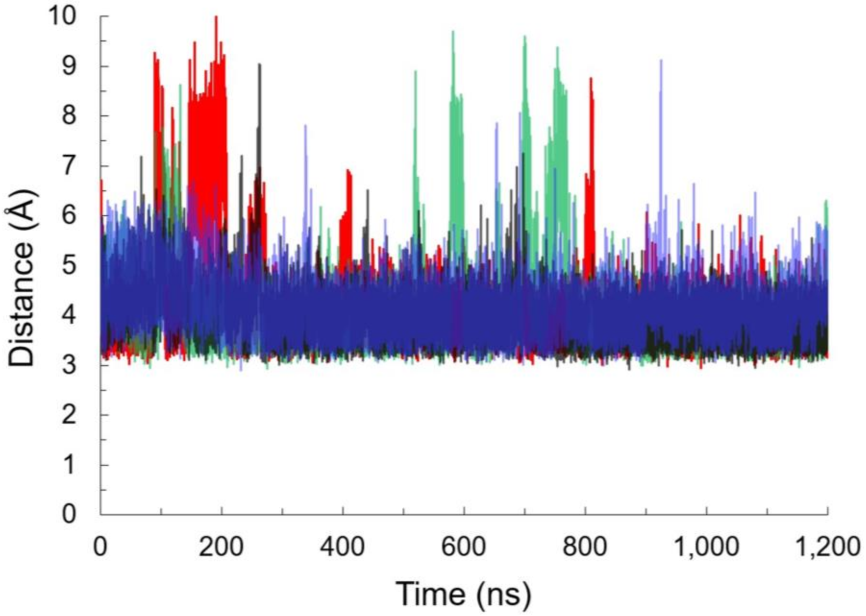

Figure S10. The distance between ligand C36 and N2 of G8 of the 5’ tetrad. Red, black, blue, and green represent four independent simulations. The first 200 ns were equilibration.
